# Supplementary material for: Single-cell transcriptome of the mouse retinal pigment epithelium in response to a low-dose of doxorubicin
Source: Commun Biol. 2022 Jul 20;5:722. doi: 10.1038/s42003-022-03676-3 (PMC9300683; doi:10.1038/s42003-022-03676-3)
Supplement: Supplementary file 2 — Supplemenatry information [file 42003_2022_3676_MOESM2_ESM.pdf]

Supplementary Information

for

Single-cell transcriptome of the mouse retinal pigment epithelium in response to a low-dose of doxorubicin

Supplementary Table 1. Representative marker genes of each cluster in control RPE cells sorted by fold changes

Control	Gene	Fold change	% of cells in cluster	% of cells not in cluster	Adjusted p value
Cluster 1	Mt3	0.865	1	0.898	3.21E-74
	Rgr	0.730	1	9.97E-01	3.59E-92
	Ttr	0.730	1	1.00E+00	3.28E-76
	Enpp2	0.711	0.978	9.27E-01	6.08E-35
	Rd3l	0.708	0.947	7.45E-01	1.2E-48
	Cltrn	0.699	0.97	8.15E-01	1.36E-63
	Mt1	0.682	1	9.94E-01	1.46E-50
	Rlbp1	0.680	1	9.58E-01	2.45E-88
	Dapl1	0.675	0.817	5.64E-01	6.9E-40
	Mt2	0.662	0.993	8.35E-01	5.81E-42
	Rdh5	0.658	0.999	9.79E-01	1.97E-84
	Rsph10b	0.658	0.925	7.11E-01	1.72E-41
	Rbp1	0.654	1	9.67E-01	5.09E-64
	Pltp	0.644	0.986	8.76E-01	3.89E-48
	Pla2g5	0.621	0.947	7.64E-01	9.11E-53
	Sostdc1	0.613	0.881	7.03E-01	6.94E-24
	Mif	0.607	0.994	8.88E-01	7.62E-56
	Krt8	0.593	0.924	7.33E-01	7.24E-38
	Slc9a3r1	0.586	0.954	8.21E-01	1.07E-44
	Phgdh	0.585	0.969	8.21E-01	2.76E-43
	Taldo1	0.582	0.984	8.17E-01	4.34E-40
	Ndufv3	0.577	0.992	8.36E-01	8.94E-47
	Apoe	0.569	1	9.77E-01	5.66E-53
	Prcd	0.566	0.906	7.03E-01	4.51E-40
	Clic6	0.566	0.951	7.95E-01	2.13E-37
Cluster 2	Dcn	3.414	0.988	0.58	2.27E-137
	Igfbp4	3.138	0.982	0.56	3.79E-132
	Cebpd	3.095	0.985	0.655	2.76E-137
	Mgp	2.760	0.97	0.32	2.72E-153
	Junb	2.736	1	0.812	9.86E-140
	Serpine2	2.709	0.982	0.422	4.64E-152
	Zfp36	2.680	0.988	0.672	2.82E-130
	S100a6	2.520	0.94	0.262	9.93E-143
	Cxcl1	2.427	0.801	0.188	5.06E-122
	Igfbp5	2.212	0.997	0.782	8.42E-123
	Apod	2.187	0.767	0.155	2.55E-116
	Gsn	2.103	0.964	0.31	4.15E-153
	Klf2	2.100	0.946	0.274	2.20E-146
	Lum	2.095	0.888	0.18	8.27E-157

	Nr4a1	2.093	0.961	0.505	6.51E-116
	Cygb	2.031	0.958	0.344	1.16E-146
	Serpinf1	1.836	0.897	0.166	2.76E-162
	Ifitm3	1.833	0.97	0.428	1.58E-126
	Id3	1.819	0.997	0.791	2.66E-103
	Serping1	1.808	0.918	0.168	1.48E-170
	Myoc	1.808	0.353	0.165	4.92E-12
	Ier2	1.806	0.97	0.45	5.81E-118
	Phlda1	1.783	0.921	0.334	4.72E-111
	Edn3	1.683	0.912	0.175	4.07E-163
	Gadd45g	1.666	0.879	0.423	3.03E-87
Cluster 3	Gm26917	4.868	0.958	0.665	1.52E-88
	Malat1	3.571	1	0.998	3.10E-98
	Kcnq1ot1	2.696	0.7	0.347	4.85E-47
	Col4a4	2.410	0.934	0.801	2.88E-67
	Nktr	2.331	0.84	0.665	9.86E-52
	Neat1	2.305	0.516	0.322	3.49E-16
	Snrnp70	2.154	0.854	0.846	7.00E-46
	Srrm2	2.079	0.981	0.963	1.23E-69
	Pnisr	1.999	0.779	0.674	1.77E-36
	Pmel	1.998	0.878	0.846	1.83E-38
	Col4a3	1.970	0.77	0.667	1.28E-35
	Gm45895	1.958	0.718	0.663	6.52E-23
	Trpm3	1.915	0.995	0.998	2.46E-67
	Syne2	1.913	0.728	0.668	5.44E-25
	Appl2	1.847	0.789	0.774	7.03E-28
	Ddx17	1.807	0.714	0.658	6.39E-23
	Macf1	1.774	0.85	0.852	2.52E-41
	Pde1c	1.714	0.789	0.757	1.06E-29
	Leng8	1.709	0.657	0.514	2.05E-20
	Fosb	1.687	0.507	0.454	0.00038
	Utrn	1.673	0.831	0.803	8.38E-37
	Itpr1	1.665	0.737	0.747	2.28E-21
	Nufip2	1.654	0.587	0.475	3.51E-15
	Akap8l	1.646	0.681	0.601	6.63E-19
	Prpf4b	1.644	0.69	0.593	6.81E-22
Cluster 4	Serpina3n	2.267	0.633	0.11	3.83E-46
	Gpnmb	1.886	0.969	0.835	1.01E-20
	Trf	1.835	1	1	8.00E-40
	Ctsd	1.205	1	0.971	2.40E-18
	Cryab	1.085	1	0.944	1.03E-09
	Cox8b	1.084	0.959	0.743	1.40E-07
	Vim	1.050	0.878	0.777	0.018451
	Ctsb	0.996	1	0.95	5.31E-22

	Tsc22d4	0.989	1	0.688	1.24E-20
	Dbi	0.987	1	0.927	2.41E-19
	Gsta3	0.961	0.612	0.124	3.88E-37
	Gpx4	0.944	1	0.991	2.95E-30
	Dhrs1	0.930	0.939	0.68	6.79E-14
	Hspb6	0.916	0.847	0.384	5.42E-23
	Psmd8	0.857	0.98	0.724	8.47E-16
	Cd9	0.839	0.969	0.733	5.54E-13
	Tubb2a	0.839	0.959	0.673	5.74E-08
	Prr13	0.827	0.98	0.8	5.34E-08
	Sema3b	0.791	0.969	0.775	1.40E-13
	Pcolce	0.740	1	0.916	5.19E-09
	H2afj	0.727	1	0.87	2.22E-15
	Lalba	0.724	0.735	0.334	1.52E-12
	Serf2	0.716	1	0.935	5.23E-17
	A2m	0.702	0.969	0.781	1.60E-05
	Cox5a	0.700	0.98	0.775	9.40E-18
Cluster 5	Rgs5	4.168	1	0.22	1.47E-17
	Acta2	4.031	1	0.796	9.23E-09
	Myl9	3.799	1	0.289	9.89E-15
	Crip1	3.761	1	0.36	1.06E-11
	Myh11	3.707	1	0.093	1.24E-38
	Tagln	3.316	1	0.146	1.15E-25
	Rrad	3.076	0.889	0.079	5.56E-33
	Tpm1	2.887	1	0.596	7.73E-09
	Tpm2	2.851	1	0.188	1.86E-20
	Sparcl1	2.795	0.944	0.316	1.61E-10
	Mustn1	2.659	1	0.082	8.72E-42
	Mylk	2.526	1	0.223	7.97E-17
	Nr4a2	2.469	1	0.231	1.20E-14
	Rasl11a	2.415	0.889	0.339	9.15E-06
	2210407C18Rik	2.160	0.667	0.019	1.54E-61
	Cebpb	1.929	1	0.575	0.000158
	Atf3	1.921	0.944	0.469	0.014466
	Bcam	1.905	1	0.25	5.25E-15
	Tm4sf1	1.785	1	0.194	9.06E-16
	Btg2	1.761	1	0.562	0.003857
	Nr4a1	1.759	1	0.599	0.000374
	Ppp1r15a	1.716	1	0.693	0.00156
	Rasd1	1.678	1	0.245	3.55E-12
	Gadd45b	1.671	1	0.426	0.000162
	Flna	1.669	1	0.456	1.35E-07

The table shows the 25 genes selected as marker genes for each subpopulation of control RPE cells according to their fold changes and expression enrichment in comparison with the other subpopulations.

Supplementary Table 2. Representative marker genes of each cluster in low-dose doxorubicin-treated RPE cells sorted by fold changes

Dox	Gene	Fold change	% of cells in cluster	% of cells not in cluster	Adjusted p value
Cluster 1	Mt3	1.447	0.996	0.884	2.21E-158
	Rd3l	1.169	0.954	0.668	1.44E-123
	Enpp2	1.086	0.988	0.925	1.01E-91
	Mt1	1.060	1	0.997	2.20E-119
	Mt2	1.000	0.997	0.834	2.12E-104
	Rgr	0.923	1	1	5.75E-147
	Rsph10b	0.917	0.948	0.669	9.52E-88
	Rbp1	0.902	1	0.974	2.17E-130
	Pltp	0.859	0.995	0.896	1.60E-102
	Calml4	0.832	0.926	0.639	7.47E-84
	Ptgds	0.824	1	1	2.14E-97
	Hmgcs2	0.813	0.955	0.67	1.34E-92
	Tma7	0.812	0.964	0.709	1.02E-83
	Ndufv3	0.805	0.991	0.79	2.33E-87
	Apoe	0.797	1	0.983	8.71E-111
	Atp5k	0.783	0.965	0.725	4.33E-82
	Sostdc1	0.781	0.917	0.67	2.33E-57
	Prcd	0.777	0.919	0.672	6.74E-82
	Ttr	0.773	1	1	1.37E-95
	Dapl1	0.771	0.862	0.563	1.53E-60
	Rom1	0.737	0.914	0.696	2.59E-66
	Rdh5	0.734	1	0.987	5.34E-114
	Mif	0.726	0.997	0.904	3.96E-85
	Uqcr11	0.719	0.987	0.782	3.76E-75
	Eci1	0.718	0.951	0.743	8.04E-78
Cluster 2	Dcn	3.533	0.986	0.448	4.48E-113
	Igfbp4	3.488	0.981	0.525	2.24E-108
	Junb	2.992	0.991	0.779	2.01E-103
	Mgp	2.990	0.94	0.227	1.11E-135
	Cebpd	2.898	0.986	0.66	1.17E-93
	Serpine2	2.844	0.986	0.413	4.38E-121
	Zfp36	2.782	0.986	0.64	9.61E-97
	Cxcl1	2.605	0.787	0.155	2.60E-111
	Gsn	2.460	0.954	0.267	1.02E-132
	Nr4a1	2.371	0.954	0.448	6.90E-93
	Igfbp5	2.294	0.995	0.804	5.04E-86
	S100a6	2.277	0.912	0.195	2.30E-127
	Cygb	2.216	0.968	0.311	1.61E-122
	Klf2	2.178	0.921	0.253	2.15E-116

	Lum	2.131	0.875	0.12	5.73E-161
	Phlda1	1.967	0.884	0.247	4.67E-103
	Apod	1.904	0.699	0.122	8.93E-97
	Id3	1.896	0.995	0.771	5.27E-74
	Serpinf1	1.778	0.921	0.142	1.01E-160
	Bgn	1.769	0.94	0.273	1.96E-113
	Cebpb	1.763	0.94	0.533	4.32E-67
	Ier2	1.744	0.944	0.429	9.04E-81
	Serping1	1.738	0.94	0.15	4.52E-161
	Gadd45g	1.688	0.866	0.427	5.22E-61
	Edn3	1.683	0.884	0.12	6.37E-157
Cluster 3	Gm26917	4.984	0.964	0.624	1.79E-122
	Malat1	3.813	0.996	1	1.78E-131
	Kcnq1ot1	2.474	0.744	0.364	1.45E-70
	Neat1	2.418	0.552	0.298	9.31E-31
	Col4a4	2.356	0.906	0.845	4.00E-89
	Srrm2	2.302	0.982	0.96	5.77E-112
	Nktr	2.277	0.87	0.695	1.80E-80
	Gm45895	2.269	0.751	0.681	5.16E-43
	Snrnp70	2.259	0.924	0.868	2.99E-94
	Fosb	2.239	0.581	0.388	5.05E-26
	Macf1	1.990	0.917	0.863	1.27E-86
	Pnisr	1.948	0.834	0.683	1.08E-62
	Fus	1.919	0.881	0.872	1.92E-66
	Akap8l	1.878	0.733	0.601	1.11E-41
	Pde1c	1.867	0.816	0.802	8.51E-51
	Col4a3	1.845	0.783	0.717	3.37E-48
	Trpm3	1.841	0.996	0.995	1.30E-114
	Itpr1	1.824	0.816	0.719	1.21E-57
	Syne2	1.823	0.755	0.689	3.60E-39
	Jun	1.820	0.895	0.873	1.78E-42
	Nufip2	1.819	0.664	0.527	2.69E-33
	Son	1.794	0.975	0.973	1.40E-96
	Sfpq	1.778	0.69	0.589	2.16E-30
	Appl2	1.776	0.827	0.768	2.97E-53
	Trpm7	1.722	0.69	0.546	1.34E-36
Cluster 4	Serpina3n	3.974	0.984	0.41	7.10E-126
	Chil1	2.909	0.984	0.813	5.84E-76
	A2m	2.325	0.992	0.851	5.12E-81
	Gpnmb	2.232	1	0.769	1.10E-100
	Trf	1.980	1	1	1.68E-102
	Pcolce	1.698	0.996	0.924	2.70E-102
	Tsc22d4	1.513	0.963	0.698	7.30E-72
	Gsta3	1.513	0.861	0.162	1.59E-134

	Lalba	1.491	0.787	0.312	8.61E-59
	Ctsd	1.441	1	0.987	1.24E-85
	Vim	1.441	0.971	0.701	1.63E-39
	Cdkn1a	1.417	0.914	0.281	3.01E-95
	Ctsb	1.308	0.996	0.933	5.19E-99
	Ifitm3	1.302	0.873	0.522	3.59E-32
	Ifit3	1.164	0.881	0.549	9.15E-34
	Gas6	1.159	0.971	0.651	5.49E-71
	Phlda3	1.114	0.889	0.279	1.17E-96
	Gpx4	1.075	1	0.997	2.70E-83
	Cox8b	1.058	0.93	0.778	9.09E-23
	Timp1	1.019	0.648	0.059	2.55E-129
	Ift57	1.014	0.93	0.534	3.26E-64
	Cd9	1.009	0.951	0.699	6.54E-58
	Gnb3	1.008	0.848	0.103	2.14E-155
	Igfbp7	0.998	0.992	0.922	1.14E-51
	H2-K1	0.987	0.914	0.543	4.85E-55
Cluster 5	Rgs5	4.742	1	0.128	3.09E-08
	Crip1	4.534	1	0.228	0.000616
	Myl9	4.256	1	0.168	0.000008
	Tagln	3.710	1	0.094	1.20E-11
	Myh11	3.426	1	0.051	6.68E-22
	Mustn1	3.316	1	0.056	6.20E-20
	Tpm2	3.267	1	0.142	2.85E-07
	Rrad	3.233	1	0.057	4.22E-20
	Sparcl1	2.873	1	0.125	4.73E-08
	Mylk	2.724	1	0.118	6.91E-09
	Rasl11a	2.596	1	0.29	0.009746
	2210407C18Rik	2.570	1	0.011	1.22E-86
	Tm4sf1	2.407	1	0.104	3.69E-10
	Rasd1	2.402	1	0.175	5.48E-05
	Tesc	2.168	1	0.007	3.13E-120
	Pde3a	1.974	1	0.071	5.60E-15
	Des	1.916	0.833	0.065	2.43E-10
	Nr4a2	1.900	0.833	0.144	0.00406
	Rbpms	1.891	1	0.226	0.002253
	Rcan2	1.868	1	0.084	3.52E-13
	Cox4i2	1.801	1	0.18	1.93E-05
	Tinagl1	1.800	0.833	0.025	7.36E-30
	Cavin3	1.726	1	0.192	0.000737
	Fxyd1	1.697	1	0.17	0.000299
	Filip1l	1.695	0.833	0.018	6.22E-40

The table shows the 25 genes selected as marker genes for each subpopulation of doxorubicin-treated RPE cells according to their fold changes and expression enrichment in comparison with the other subpopulations.

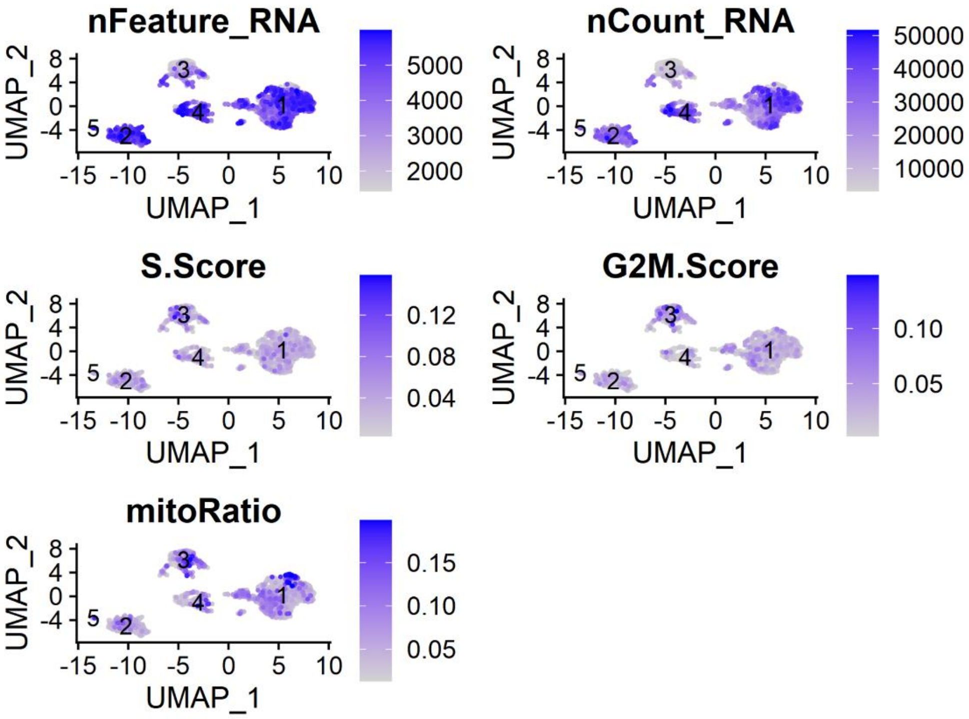

Supplementary Fig. 1. UMAP features for the potential confounding factors affecting the cluster number. The UMAP feature plots of parameters including nFeature_RNA, nCount_RNA, S.Score, G2 M.Score and mitoRatio are presented to verify that the dataset was classified into an appropriate number of clusters and to confirm that there were other factors that could affect the formation of clusters other than gene expression. These parameters do not show bias toward a specific cluster, so we determined that the clustering result in the corresponding cell data was appropriate.

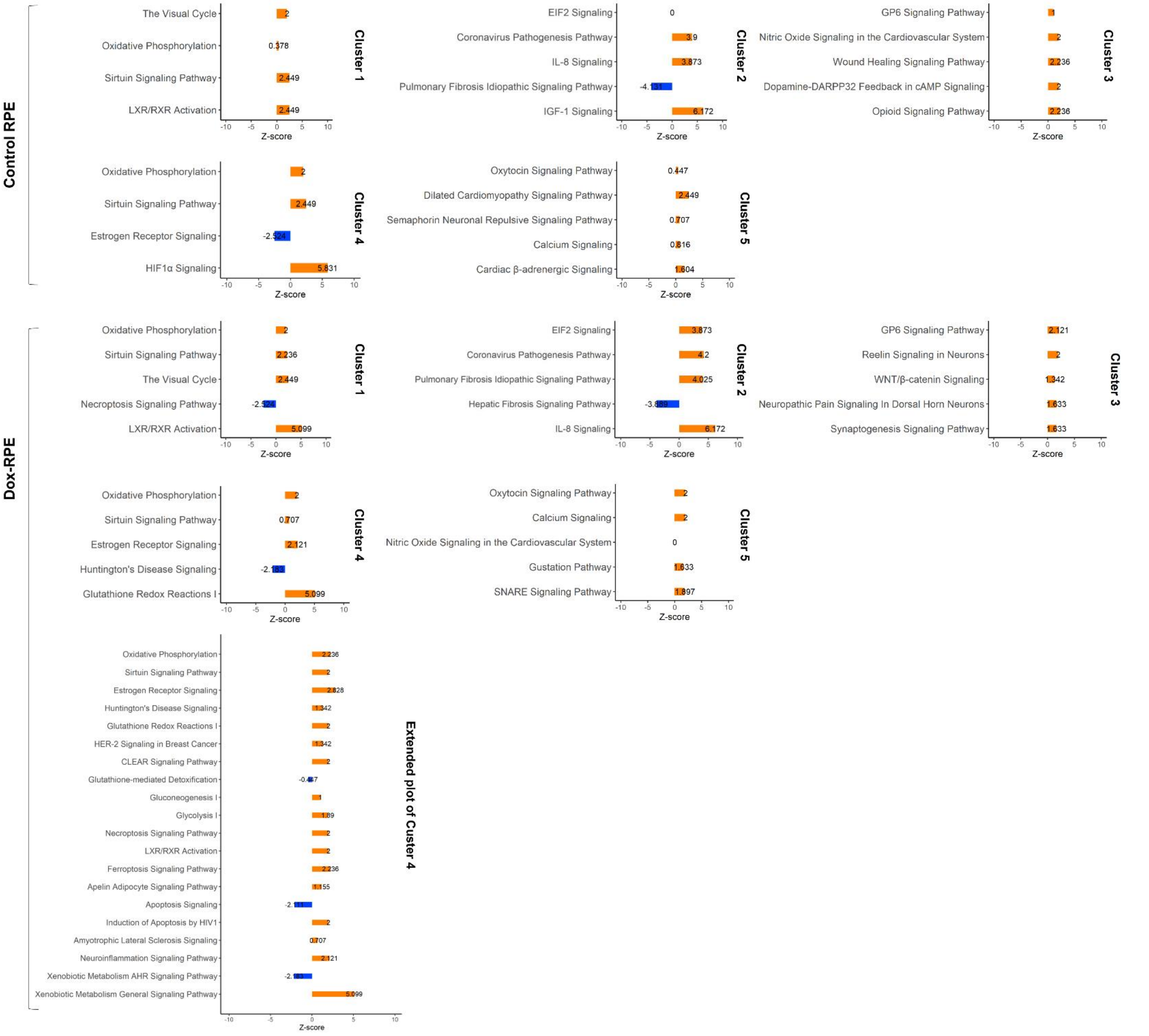

Supplementary Fig. 2. Canonical pathway analysis using Ingenuity Pathway Analysis (IPA). The top 5 canonical pathways for each cluster of control RPE cells and Dox-RPE cells are represented. The 5 subclusters showed distinct pathways, and general overlap of the pathways was found between the control- and Dox-RPE clusters. The apoptosis pathway was only found in cluster 4 of the Dox-RPE cells (extended plot of cluster 4).

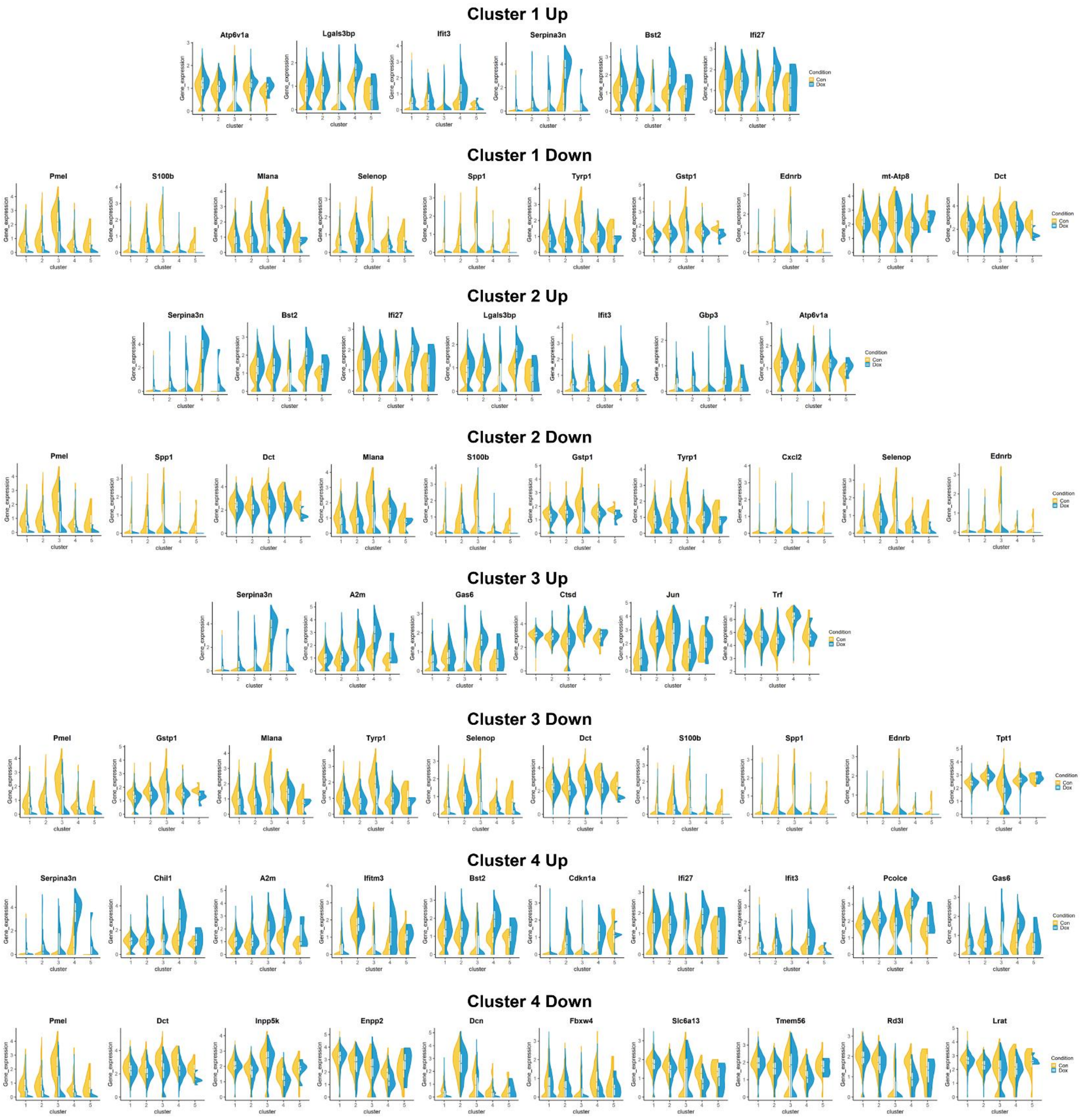

Supplementary Fig. 3. Representative up-regulated and down-regulated DEGs after doxorubicin treatment in 4 RPE clusters. DEG of cluster 5 was not depicted because no genes were significantly different between control and Dox-RPEs. The top 10 genes were selected, but fewer than 10 up-regulated DEGs are shown because they are the only significant DEGs. Each fold change between Con- and Dox-RPE cells in all up- or down-regulated DEGs in the 4 RPE clusters was statistically significant.

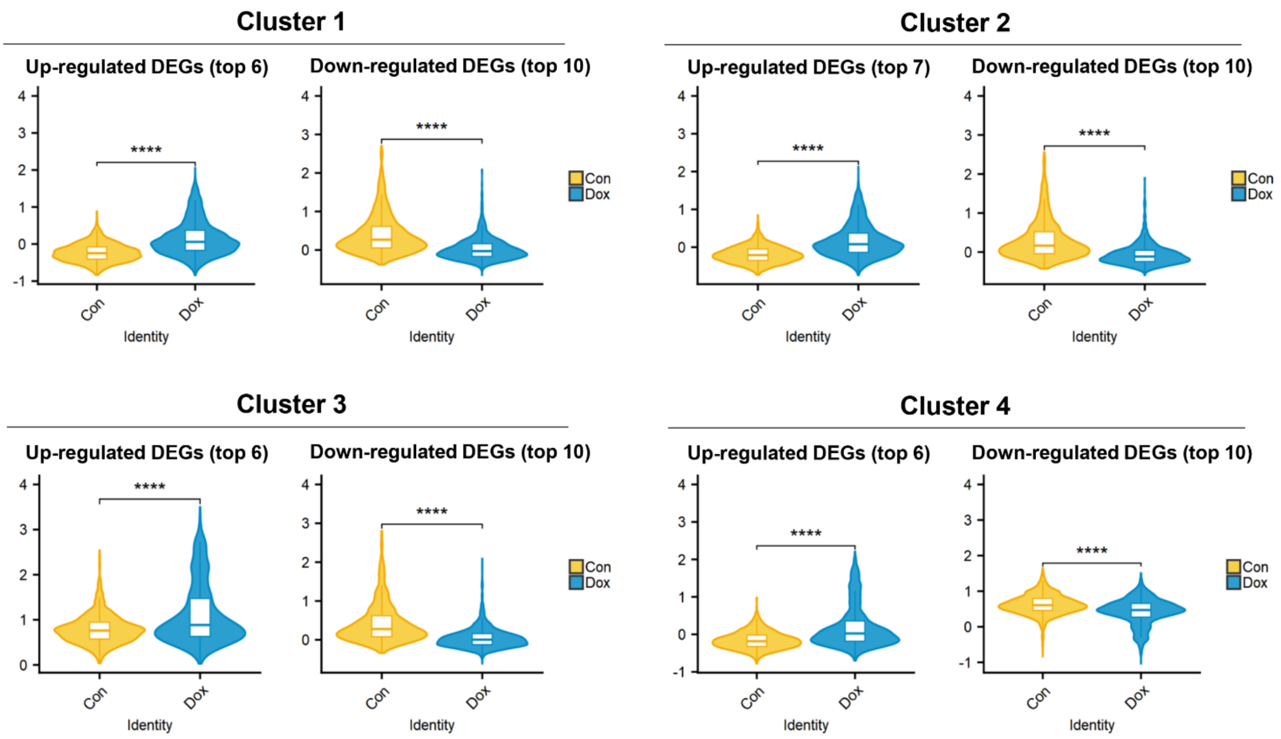

Supplementary Fig. 4. Comprehensive expression of all upregulated and downregulated DEGs after doxorubicin treatment per cluster. Comprehensive expression was calculated using the AddModuleScore() function in Seurat to represent the difference in expression by condition. DEG of cluster 5 was not depicted because no genes were significantly different between control and Dox-RPEs. The top 10 genes were selected, but fewer than 10 up-regulated DEGs are shown because they are the only significant DEGs. (****p<0.0001)

a

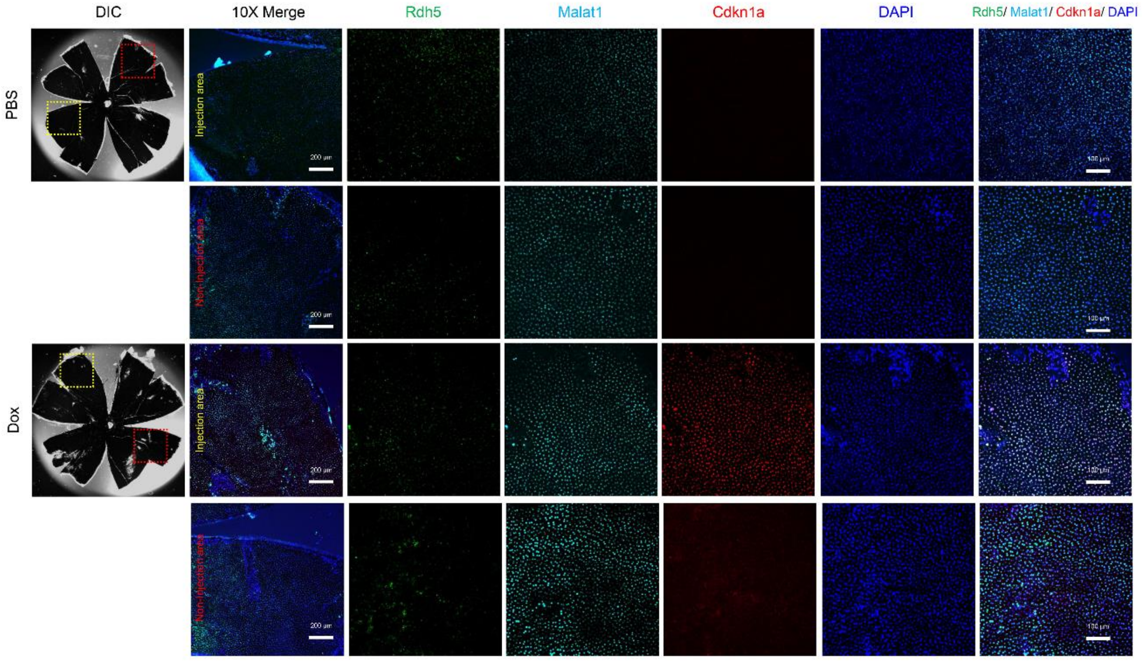

PBS injected area

Rdh5	Malat1** 0	1
0	4	23
1	7	30

Rdh5	Cdkn1*** 0	1
0	28	1
1	24	13

Malat1	Cdkn1*** 0	1
0	8	3
1	42	11

PBS noninjected area

Rdh5	Malat1*** 0	1
0	0	30
1	1	33

Rdh5	Cdkn1*** 0	1
0	29	1
1	22	12

Malat1	Cdkn1*** 0	1
0	1	0
1	50	13

Dox injected area

Rdh5	Malat1*** 0	1
0	6	30
1	5	23

Rdh5	Cdkn1*** 0	1
0	0	36
1	0	28

Malat1	Cdkn1*** 0	1
0	0	11
1	0	53

Dox noninjected area

Rdh5	Malat1*** 0	1
0	4	20
1	0	40

Rdh5	Cdkn1*** 0	1
0	24	0
1	30	10

Malat1	Cdkn1*** 0	1
0	4	0
1	50	10

b

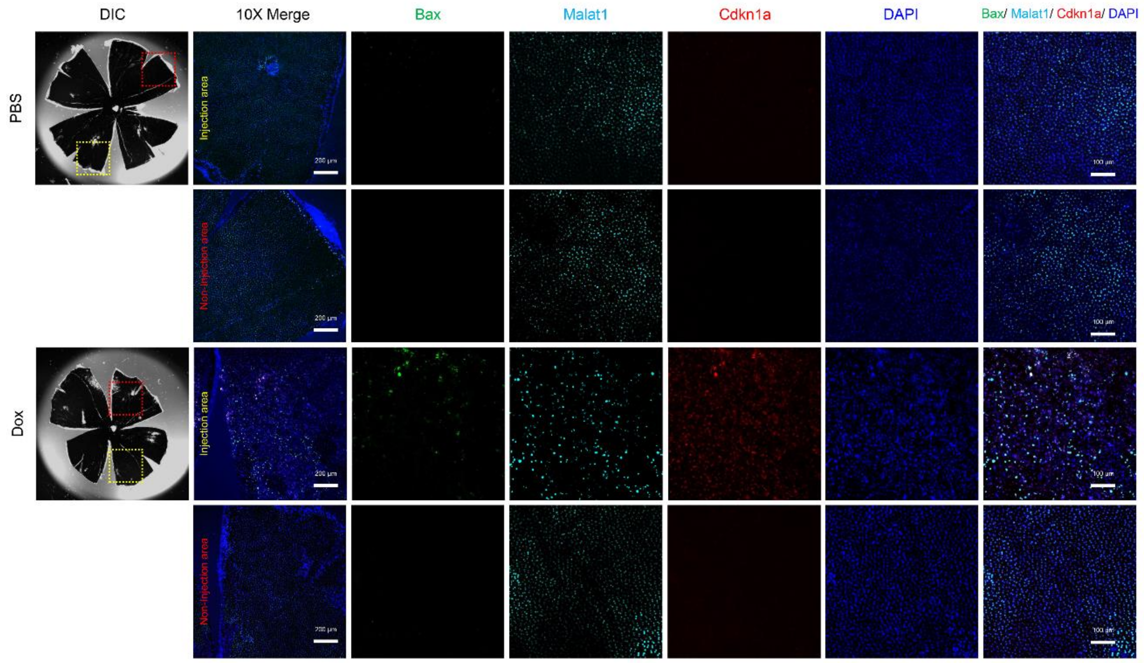

PBS injected area

Bax	Malat1***	
	0	1
0	0	28
1	0	36

Bax	Cdkn1	
	0	1
0	27	1
1	0	36

Malat1	Cdkn1***	
	0	1
0	0	0
1	27	37

PBS noninjected area

Bax	Malat1***	
	0	1
0	0	55
1	0	9

Bax	Cdkn1	
	0	1
0	53	2
1	2	7

Malat1	Cdkn1***	
	0	1
0	0	0
1	55	9

Dox injected area

Bax	Malat1***	
	0	1
0	0	25
1	0	39

Bax	Cdkn1	
	0	1
0	25	0
1	0	39

Malat1	Cdkn1***	
	0	1
0	0	0
1	25	39

Dox noninjected area

Bax	Malat1***	
	0	1
0	0	58
1	0	6

Bax	Cdkn1	
	0	1
0	48	10
1	3	3

Malat1	Cdkn1***	
	0	1
0	0	0
1	51	13

c

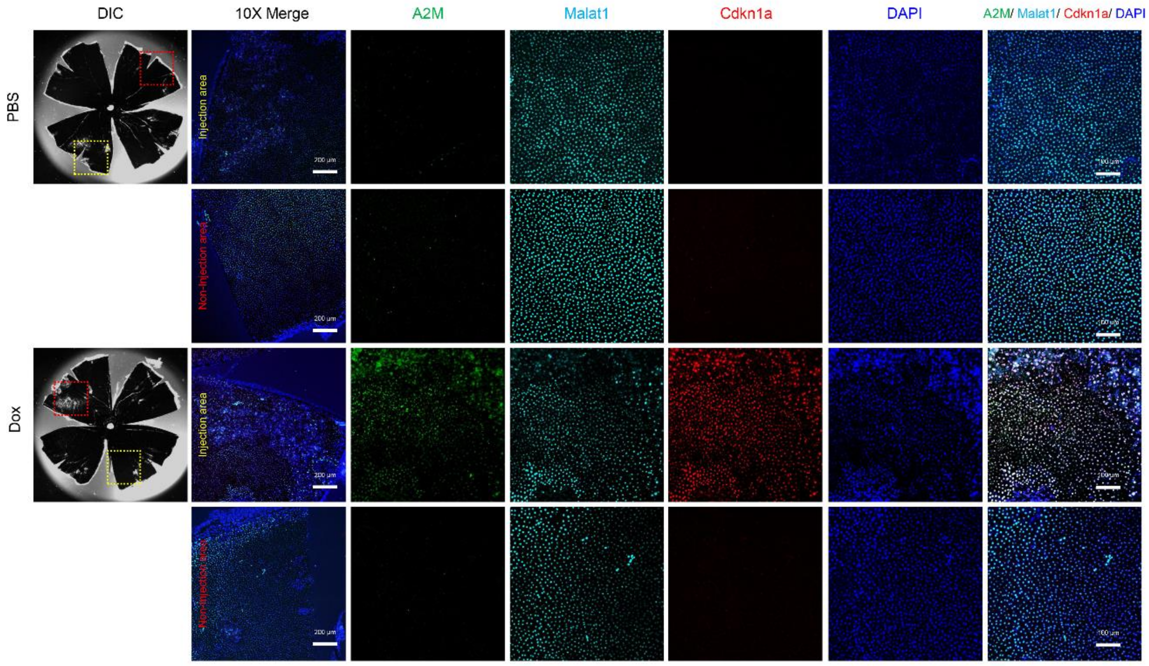

PBS injected area

A2M	Malat1***	
	0	1
0	1	19
1	0	44

A2M	Cdkn1	
	0	1
0	18	2
1	8	36

Malat1	Cdkn1***	
	0	1
0	1	0
1	25	38

PBS noninjected area

A2M	Malat1***	
	0	1
0	0	43
1	0	21

A2M	Cdkn1	
	0	1
0	40	3
1	2	19

Malat1	Cdkn1***	
	0	1
0	0	0
1	42	22

Dox injected area

A2M	Malat1**	
	0	1
0	0	3
1	15	46

A2M	Cdkn1	
	0	1
0	0	3
1	0	61

Malat1	Cdkn1***	
	0	1
0	0	15
1	0	49

Dox noninjected area

A2M	Malat1***	
	0	1
0	8	48
1	0	8

A2M	Cdkn1	
	0	1
0	56	0
1	1	7

Malat1	Cdkn1***	
	0	1
0	8	0
1	49	7

Supplementary Fig. 5. RNA fluorescence in situ hybridization for the combination of RPE subpopulations in control RPE and Dox-RPE cells**. a** Images showing mRNA expression of *Rdh5* (green), *Malat1* (cyan), and *Cdkn1a* (red), which are selected markers for RPE subpopulations 1, 3, and 4, respectively. Nuclei were counterstained with DAPI. Differential interference contrast (DIC) images represent the whole shape of the RPE flatmount and the inspected location in the injected area (yellow box) and noninjected area (red box). Below the fluorescence images, 2 x 2 contingency tables are listed between 2 markers in four environments of the same RPE flatmount in PBS-injected/noninjected areas and Dox-injected/noninjected areas. Statistical analyses were performed using McNemar's test (*p < 0.05, **p < 0.01, ***p < 0.001). Scale bar: 200 μm for 10X merge image (first column) and 100 μm for 20X magnification (next columns on the right side). **b** Images and 2 x 2 contingency tables for the comparison of spatial distribution of showing mRNA expression of *Bax* (green), *Malat1* (cyan) and *Cdkn1a* (red), which are selected markers for RPE subpopulations 4, 3, and 4, respectively. **c** Images and 2 x 2 contingency tables for the comparison of spatial distribution showing mRNA expression of *A2M* (green), *Malat1* (cyan), and *Cdkn1a* (red), which are selected markers for RPE subpopulations 4, 3, and 4, respectively. PBS=RPE flatmount from the PBS-injected mouse; Dox=RPE flatmount from the Dox-injected mouse.
