# Supplementary material for: Single-cell transcriptome of the mouse retinal pigment epithelium in response to a low-dose of doxorubicin
Source: Commun Biol. 2022 Jul 20;5:722. doi: 10.1038/s42003-022-03676-3 (PMC9300683; doi:10.1038/s42003-022-03676-3)
Supplement: Supplementary file 3 — Description of Additional Supplementary Files [file 42003_2022_3676_MOESM3_ESM.pdf]

Description of Additional Supplementary Files

File name: Supplementary Data 1
Description: Gene expression signatures and suggested major cell types of each cluster.

File name: Supplementary Data 2
Description: Comprehensive representation of gene ontology processes in the control RPE.

File name: Supplementary Data 3
Description: Comprehensive representation of gene ontology processes in the doxorubicin-treated RPE.

File name: Supplementary Data 4
Description: Significant DEGs in any of the 5 clusters between control and doxorubicin-treated RPE.
